# The autonomic and nociceptive response to acute experimental stress is impaired in people with knee osteoarthritis: A preliminary study

**DOI:** 10.1016/j.ynpai.2023.100144

**Published:** 2023-09-20

**Authors:** Neil R. Bossenger, Gwyn N. Lewis, David A. Rice, Daniel Shepherd

**Affiliations:** a Health and Rehabilitation Research Institute, Auckland University of Technology, Auckland, New Zealand; b Waitematā Pain Service, Te Whatu Ora Waitematā, Auckland, New Zealand; c Department of Psychology, Faculty of Health and Environmental Sciences, Auckland University of Technology, Auckland, New Zealand

**Keywords:** Knee osteoarthritis, Fibromyalgia, Autonomic nervous system, Mental arithmetic, Thermal pain

## Abstract

**Objective:**

Alterations in autonomic function are evident in some chronic pain conditions but have not been thoroughly examined in people with osteoarthritis (OA). The study aimed to examine resting autonomic nervous system (ANS) function in people with knee OA, and the response of the autonomic and nociceptive systems to acute stress.

**Methods:**

A preliminary cross-sectional study was undertaken involving people with knee OA (*n* = 14), fibromyalgia (*n* = 13), and pain-free controls (*n* = 15). The sympathetic and parasympathetic components of the ANS were assessed through measures of pre-ejection period (PEP), skin conductance level (SCL), and high frequency heart rate variability (HF HRV). The nociceptive system was assessed through pain ratings associated with a tonic heat pain stimulus. In separate sessions, ANS and heat pain measures were assessed at rest and in response to nociceptive and mental arithmetic stressors.

**Results:**

The knee OA group showed reduced HF HRV at rest and reduced modulation in response to stress. Resting PEP and SCL were normal in the knee OA group but PEP modulation was impaired in both chronic pain groups during nociceptive stress. The expected reduction in tonic heat pain ratings in response to stress was lacking in the knee OA and FM groups.

**Conclusion:**

Preliminary evidence shows impaired parasympathetic nervous system function at rest and in response to nociceptive and mental stress in people with knee OA, with some evidence of altered sympathetic nervous system function. Impaired ANS function could contribute to ongoing pain experienced, and interventions that target ANS function could be beneficial.

## Highlights

• We show altered function of the autonomic nervous system in people with knee osteoarthritis, both at rest and in response to acute nociceptive and mental stress.
• Modulation of the nociceptive system in response to acute stress was also diminished in people with knee osteoarthritis.
• Interventions that target autonomic function should be investigated further in people with knee osteoarthritis.

## 1 Introduction

Knee osteoarthritis (OA) is characterised by joint pain, stiffness, and impaired physical function (Hunter et al., 2014). While total knee arthroplasty (TKA) is recommended for end-stage knee OA that is not responsive to conservative treatment, the expected increase in the number of TKA undertaken (Sloan et al., 2018) and relatively high rate of patient dissatisfaction with the outcome (Baker et al., 2013) suggest alternative conservative treatment methods are warranted.

People with other chronic pain conditions, including fibromyalgia (FM) (Cohen et al., 2001, Reyes del Paso et al., 2011, Desmeules et al., 2003), rheumatoid arthritis (Drummond and Willox, 2013, Meeus et al., 2012); and complex regional pain syndrome (CRPS) (Terkelsen et al., 2012) demonstrate alterations in both nociceptive processing and autonomic nervous system (ANS) function. These two systems are interlinked both anatomically, through shared brain structures, and functionally, in their role of adapting to threat and modulating pain (Benarroch, 2006). For example, activation of the parasympathetic nervous system is associated with facilitation of brainstem regions involved in mediating descending nociceptive inhibition (Benarroch, 2006). Vagal activity also regulates immune reactivity via cholinergic anti-inflammatory pathways, inhibiting the release of proinflammatory cytokines (Courties et al., 2017), which can reduce nociceptive sensitisation. Therefore, dysfunction in the ANS may impact both the resting function and the modulatory capacity of the nociceptive system.

The ANS is critical in responding to stress. Exposure to acute stress elicits both beta adrenergic sympathetic activation and vagal withdrawal (Brindle et al., 2014). The consequence of this tends to be increased heart rate, blood pressure, and electrodermal activity, and increased levels of plasma catecholamines (Kyle and McNeil, 2014, McDougall et al., 2005). Functional deficits in the ANS response to stress can lead to diminished cardiovascular regulation (Reyes del Paso et al., 2010, Thieme et al., 2006) and reduced blood pressure-mediated baroreceptor activation, which may contribute to a decrease in descending nociceptive inhibition and an impaired ability to modulate nociception under conditions of stress (Drummond and Willox, 2013, Reyes del Paso et al., 2010, Chalaye et al., 2013, Bruehl et al., 2010). In support of this, studies examining the nociceptive effects of acute mental stress in people with FM (Drummond and Willox, 2013, Reyes del Paso et al., 2010), rheumatoid arthritis (Drummond and Willox, 2013), and CRPS (Drummond et al., 2001) have revealed that mental stress may increase pain, in contrast to stress-induced hypoalgesia that is normally evident in healthy populations (Drummond et al., 2001). Similarly, a reduced cardiovascular response to acute nociceptive stress has been shown in people with chronic pain compared to healthy populations (Reyes del Paso et al., 2010, Chalaye et al., 2014).

Only one previous study has investigated resting ANS function in people with knee OA (Bossenger et al., 2023), reporting reduced parasympathetic activity at rest and an impaired autonomic and nociceptive response to acute exercise. No other studies have directly examined how acutely stressful stimuli impact the autonomic and nociceptive systems in people with OA. Therefore, the goal of the current study was to examine ANS function in people with knee OA at both rest and in response to acute nociceptive and mental stressors. A further group of people with FM were included as altered autonomic function has been shown in this population previously. It was hypothesised that people with knee OA and FM would show reduced parasympathetic and increased sympathetic activity at rest. It was further hypothesised that the modulation of the ANS in response to stress would be diminished in both chronic pain populations, and that this would be accompanied by impaired nociceptive inhibition.

## 2 Methods

### 2.1 Participants

A sample size calculation was undertaken using an alpha level of 0.05 and power of 0.8 (G*Power 3.1.9.2). An effect size of 0.72, based on a previous study assessing high frequency heart rate variability (HF HRV) in people with CRPS in response to stressful mental arithmetic (Terkelsen et al., 2012), indicated 25 participants were required per group. However, during the period of recruitment, COVID-19 led to several lockdown periods in the recruitment region. As such, only 42 (15 control, 14 knee OA, 13 FM) of the planned 75 participants were able to be recruited and we have therefore treated the findings as preliminary. These were the same participants included in Bossenger et al. (Bossenger et al., 2023); however, the data in the current study were collected separately.

Participants were included in the knee OA or FM groups if they were aged over 18 years, had been diagnosed by a clinician and fulfilled the American College of Rheumatology criteria for OA or FM (Altman et al., 1986, Wolfe et al., 2016), and had experienced pain for at least 3 months on most days with a minimum level of 3 out of 10 in the previous 7 days. Control participants were required to be aged over 18 years, currently pain-free, and without a history of chronic pain. Participants were excluded if they had a cardiac condition, hypertension (i.e., systolic blood pressure > 140 mmHg and/or diastolic blood pressure > 90 mmHg), or were taking medications that may alter cardiovascular and/or autonomic activity. Participants were asked to refrain from taking analgesic medication for 24 hrs prior to data collection and from consuming caffeine and tobacco products 6 hrs prior to data collection. Ethical approval was obtained from the institutional ethics committee and participants provided informed, written consent prior to participation.

### 2.2 Procedure

**Fig. 1:**
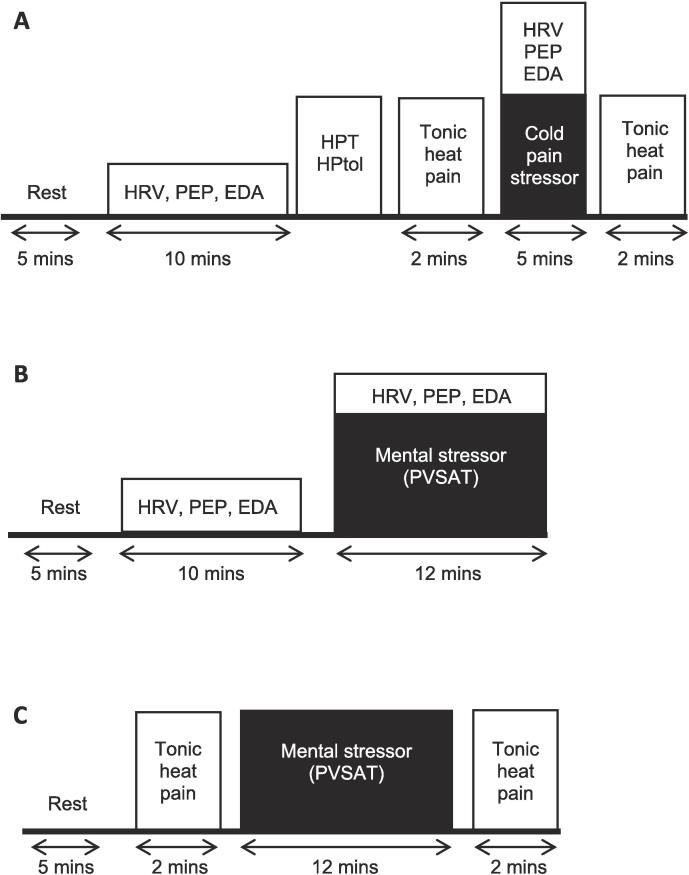
Schematic diagram of the three testing sessions. The first session (top) involved assessment of the nociceptive stressor (cold pain). Autonomic nervous system (ANS) outcome measures were collected for 10 mins at rest and during the 5 min stressor. Tonic heat pain ratings were obtained before and immediately after the 5 min stressor. The second and third sessions were randomised and assessed the ANS (middle) and nociceptive (bottom) response to the mental stressor using similar protocols to the first session. HRV = heart rate variability; PEP = pre-ejection period; EDA = electrodermal activity; HPT = heat pain threshold; HPtol = heat pain tolerance; PVSAT = paced visual serial addition task.

The study was a cross-sectional, experimental design undertaken in a laboratory setting within a public hospital. Data collection was conducted over three separate sessions separated by at least 7 days (see Fig. 1). At the first session, participants completed clinical and psychosocial questionnaires and the response to the nociceptive stressor was assessed. The questionnaires consisted of the Brief Pain Inventory (BPI), Pain Catastrophising Scale (PCS), Depression, Anxiety, and Stress Scale (DASS-21), and the Western Ontario and McMaster Universities Osteoarthritis Index (WOMAC; OA group only). To assess the response to the nociceptive stressor, participants reclined in the supine position with the torso elevated at 30° for 5 mins. The ANS outcome measures were then recorded continuously for 10 mins while resting (baseline). Following this, heat pain threshold (HPT) and tolerance (HPtol) were determined and the tonic heat pain stimulus applied continuously for 2 mins. The nociceptive stressor (cold water) was then delivered for 5 mins. This was immediately followed by application of the tonic heat pain stimulus for a further 2 mins. The ANS outcome measures were continuously recorded during this time.

The remaining two sessions assessed the response to the mental stressor and were completed in a random order. ANS data were recorded in one of the sessions and tonic heat pain data in the other; these were separated due to practicality reasons of assessing both sets of outcome measures while undertaking the mental stressor and because tonic heat pain testing may induce changes in the ANS measures that would interfere with our ability to assess the effects of mental stress on ANS function. In the ANS session, the participants were positioned as described above and completed 5 mins of rest followed by 10 mins of resting ANS recording. The participants then completed the mental stressor (mental arithmetic) for 12 mins while ANS data were continuously recorded. In the tonic heat pain session, after 5 mins of rest the tonic heat pain stimulus was applied for 2 mins before and after completing the 12 min mental stressor.

### 2.3 Nociceptive stressor

Cold water was used as the nociceptive stressor. Participants immersed their arm (contralateral to the involved knee in the knee OA group, right arm for the FM/control groups) up to the wrist in 12 °C cold water for 5 mins and were requested not to move or contract their arm during immersion. If the participant was not able to withstand the cold pain, they were permitted to move their hand in and out of the water for up to 10 s, as required, in order to sustain 5 mins. Participants were instructed to rate the cold pain intensity continuously using a computerised visual analogue scale (COVAS) from 0 to 100.

### 2.4 Mental stressor

Mental arithmetic was used as the mental stressor. The paced visual serial addition test (PVSAT) is a computerised (LabVIEW; National Instruments, USA), visual version of the paced auditory serial addition test (PASAT). The program presented a random series of numbers from 1 to 9 to the participant on a screen and they were instructed to consecutively sum pairs of numbers such that each number was added to the one immediately preceding it. A computer mouse was used to select the correct answer on a keypad presented on the computer screen. The PVSAT consisted of 4 trials with a constant interstimulus interval of 2.4 s between digit presentation. Each trial involved 61 items (60 responses) according to the standard version of the PASAT (Gronwall and Wrightson, 1974). The interstimulus interval remained constant, rather than incrementally decreasing across each trial, because the PVSAT is more difficult with a mouse-click response than the verbal response of the original PASAT. A 60 s rest interval was provided between trials, giving rise to a total time of 12 mins.

### 2.5 Tonic heat pain

The nociceptive system was assessed using pain ratings in response to a tonic heat pain stimulus. Heat was delivered using a 30 × 30 mm thermode (Pathway Model ATS, Medoc, Israel) applied to the volar aspect of the forearm contralateral to that placed into the cold water. To determine HPT, the thermode temperature was initially set at 32 °C and slowly increased at a rate of 0.3 °C/s. Participants were instructed to push a response button when the sensation changed from heat to pain. To determine HPtol, the same procedure was followed but participants were instructed to respond when the pain produced by the thermode became unbearable. The procedure was performed three times for each assessment with a 30 s interval between stimuli. The average of the three assessments was used for further analysis.

The tonic heat pain test stimulus was applied for 2 mins at a constant temperature that elicited a mean pain intensity of 60 out of 100 at baseline. The temperature of the stimulus was determined as: HPT + ([Hptol – HPT]/2) (Chalaye et al., 2013). If a participant’s pain rating was not 60 ± 10 at this temperature, the temperature was adjusted up or down by 0.5 °C increments until a rating of 60 ± 10 was achieved. Participants rated their pain continuously using the COVAS, ranging from 0 (no pain) to 100 (worst pain imaginable). Tonic heat pain ratings were recorded at baseline and immediately following the nociceptive/mental stressor.

### 2.6 Autonomic outcome measures

#### 2.6.1 Heart rate variability

HF HRV data were used as a measure of parasympathetic activity as frequency domain analysis is recommended over time domain for short-term recordings of HRV (TFESCNASP Task Force of the European Society of, 1996). Continuous electrocardiogram (ECG) recordings were obtained from pregelled 46 × 88 mm Ag/AgCl ICG/ECG electrodes (Cardio Vascular Lab, Medis, Germany). A standard 8 electrode set-up was used with electrodes placed at each side of the participant’s neck (two pairs) and on each side of the thorax along the midaxillary line (two pairs) (Parry and McFetridge-Durdle, 2006). The ECG signal was sampled at 200 Hz and time locked to the R wave. The variability of RR intervals was examined using Kubios HRV Premium v3.5.0 (Kubios Oy, Finland), which includes automatic artefact rejection. Following fast-fourier transform, HF HRV (0.15–0.4 Hz) was extracted from the data obtained over the final 5-minutes of baseline and during the stressors. HF HRV data derived from 5-minute recordings have been shown to be reliable and valid measures (Schroeder et al., 2004, Sinnreich et al., 1998).

#### 2.6.2 Cardiac pre-ejection period

Pre-ejection period (PEP) was used as a measure of central sympathetic activity. PEP data were extracted from the same impedance cardiography (ICG) and ECG recording used for HRV. PEP was defined as the interval between the onset of electrical stimulation of the ventricles (Q wave on the ECG) and aortic valve opening (B point on the ICG) (Burgess et al., 2004), which has been shown to be a reliable procedure (Parry and McFetridge-Durdle, 2006). The mean PEP of the final 30 s of each minute of the recordings was determined at baseline and during the stressors.

#### 2.6.3 Electrodermal activity

Electrodermal activity (EDA) was used as a measure of peripheral sympathetic activity. EDA was recorded by placing a pair of 6 cm diameter pregelled Ag/AgCl electrodes (Red Dot, 3 M) on the palmer tips of the index and middle fingers of the hand (Waters et al., 1987), at least 10 cm away from any region on the forearm receiving thermal stimulation. Data were sampled at 32 Hz using a NeXus-10 MKII and BioTrace software (MindMedia, Netherlands). Tonic EDA in the form of skin conductance level (SCL) was assessed by determining the mean amplitude of the EDA signal during the final 10 s of each minute during the recordings at baseline and during the stressors (Treister et al., 2012). Such SCL recordings have shown moderate-excellent reliability at rest and in response to stimuli (Waters et al., 1987, Jang et al., 2019).

### 2.7 Statistical analyses

Given the small sample size, where assumptions related to the normal distribution of data are difficult to adequately assess (Altman et al., 1983), non-parametric statistics were used to analyse the data. Demographic and clinical characteristics of the three groups were compared using Kruskal-Wallis tests and the Chi-square test, as appropriate. Significant findings were followed up using the Mann-Whitney *U* test to compare the knee OA and FM groups to the control group.

Baseline ANS outcome measures and tonic heat pain ratings were averaged across the two sessions and compared among the three groups using Kruskall-Wallis tests. Significant findings were followed up with Mann-Whitney U tests to compare the knee OA and FM groups to the control group. To determine the effect of the two stressors on outcome measures, Wilcoxon Signed Ranks tests were used to compare the outcomes at baseline to during (ANS outcome measures) or following (tonic heat pain ratings) the stressor within each group. The alpha level for all statistical procedures was set to 0.05. All statistical analyses were performed using SPSS v28 (Armonk, NY: IBM Corp).

## 3 Results

### 3.1 Participant characteristics

Table 1 shows demographic and clinical information of the three groups. There were no significant differences in age, gender, or self-reported ethnicity in the two chronic pain groups compared to the control group. BPI severity, BPI interference, DASS-21, and PCS values were all significantly higher in the knee OA and FM groups compared to control (all *p* < 0.001). Two control participants exhibited previously undiagnosed cardiovascular anomalies; thus, their HR HRV data were omitted from the analyses.

**Table 1:** Participant characteristics. Data are mean ± standard deviation unless otherwise stated.

**Characteristic**	**Control** ***n* = 15**	**Knee OA** ***n* = 14**	**FM** ***n* = 13**	***p* value**
Age (years)	53 ± 10	60 ± 9	47 ± 14	0.03
Gender female (n, %)	11 (73)	10 (71)	13 (1 0 0)	0.11
Ethnicity (n, %)				0.72
European	13 (87)	12 (86)	11 (85)	
Māori or Pasifika	1 (7)	2 (14)	0 (0)	
Other	1 (7)	0 (0)	2 (15)	
BMI (kg/m^2^)	25 ± 3	31 ± 7*	31 ± 5*	0.002^#^
BPI severity	2 ± 3	18 ± 10*	20 ± 7*	<0.001^#^
BPI interference	1 ± 1	30 ± 19*	36 ± 19*	<0.001^#^
DASS-21 Depression	1 ± 1	4 ± 3*	6 ± 4*	<0.001^#^
DASS-21 Anxiety	1 ± 1	3 ± 5	6 ± 4*	<0.001^#^
DASS-21 Stress	2 ± 2	5 ± 4*	9 ± 5*	<0.001^#^
PCS	6 ± 7	17 ± 13*	22 ± 11*	<0.001^#^
WOMAC		44 ± 23		
Cold stressor pain (0–100)	56 ± 21	68 ± 25	64 ± 27	0.45
PVSAT score ANS session	56 ± 26	35 ± 20*	38 ± 22*	0.049^#^
PVSAT score THP session	63 ± 24	43 ± 25	50 ± 23	0.08

BMI = body mass index; BPI = Brief Pain Inventory; DASS-21 = Depression, Anxiety and Stress Scales; FM = fibromyalgia; OA = osteoarthritis; PCS = Pain Catastrophising Scale; SD = standard deviation; WOMAC = Western Ontario and McMaster Universities Osteoarthritis Index; ANS = autonomic nervous system; THP = tonic heat pain; PVSAT = paced visual serial addition test; * = significant difference from control; ^#^ = significant difference among groups.

### 3.2 Baseline function

Group data showing the averaged outcome measures across the two baselines are shown in Table 2. There was a significant difference in HF HRV among groups (*p* = 0.012). Follow-up Mann-Whitney U tests showed that the knee OA group had significantly lower HF HRV at baseline compared to the control group (*p* = 0.005) but the difference between the FM and control groups was not significant (*p* = 0.068).

**Table 2:** Group means ± standard deviations of the autonomic outcome measures and pain ratings at baseline.

**Variable**	**Control**	**Knee OA**	**FM**	***P* value**
HF HRV (nu)	54 ± 18	34 ± 16*	41 ± 17	0.012
PEP (ms)	115 ± 17	116 ± 26	114 ± 13	0.990
SCL (µS)	2.75 ± 1.11	3.79 ± 2.24	5.40 ± 2.86*	0.021
Tonic heat pain rating	48 ± 12	54 ± 20	45 ± 17	0.595

OA = osteoarthritis; FM = fibromyalgia; HF HRV = high frequency heart rate variability; PEP = pre-ejection period; SCL = skin conductance level. * = significant difference from control.

There was a significant difference in SCL among groups at baseline (*p* = 0.021). Follow-up Mann-Whitney U tests showed that the FM group had a significantly higher SCL compared to the control group (*p* = 0.003) but there was no difference between the knee OA and control groups (*p* = 0.35).

There were no significant differences in PEP (*p* = 0.99) or the tonic heat pain rating among groups at baseline (*p* = 0.60).

### 3.3 Nociceptive stressor

**Fig. 2:**
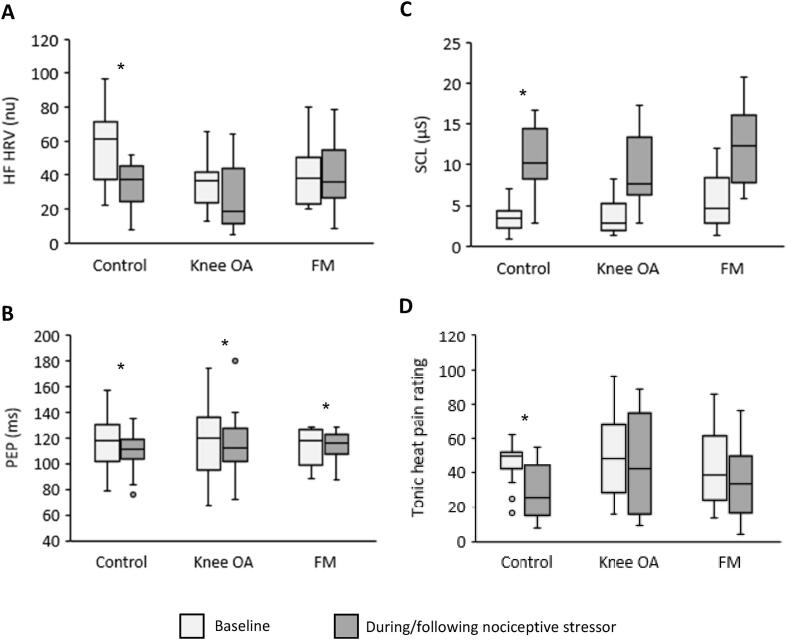
Box and whisker plots of the autonomic outcome measures and tonic heat pain ratings before and during/immediately following the nociceptive stressor. OA = osteoarthritis; FM = fibromyalgia; HF HRV = high frequency heart rate variability; PEP = pre-ejection period; SCL = skin conductance level. * = significant difference from baseline.

Group results of the response to the nociceptive stressor are shown in Fig. 2. The pain ratings (0–100) of the nociceptive stressor in the control (57 ± 21), knee OA (68 ± 25), and FM (64 ± 27) groups were not significantly different (*p* = 0.4).

There was a significant reduction in HF HRV in the control group during the stressor compared to baseline (*p* = 0.001). In contrast, HF HRV was not significantly different during the cold pain stressor compared to baseline in the knee OA (*p* = 0.06) or FM (*p* = 0.65) groups.

PEP was significantly shorter during the stressor compared to baseline in the control group (*p* = 0.02). In contrast, PEP was not significantly different during the stressor in the knee OA (*p* = 0.18) or FM groups (*p* = 0.06).

There was a significant increase in SCL in all three groups in comparison to baseline (all *p* < 0.01) during the nociceptive stressor.

There was a significant reduction in tonic heat pain rating in the control group compared to baseline (*p* = 0.001). In contrast, tonic heat pain ratings were not significantly different following the nociceptive stressor compared to baseline in the knee OA (*p* = 0.33) or FM (*p* = 0.13) groups.

### 3.4 Mental stressor

**Fig. 3:**
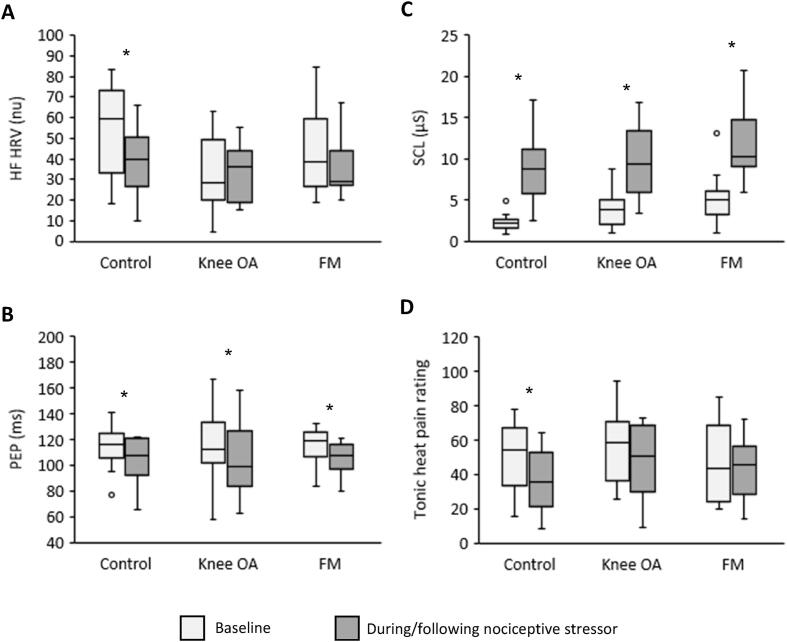
Box and whisker plots of the autonomic outcome measures and tonic heat pain ratings before and during/immediately following the mental stressor. OA = osteoarthritis; FM = fibromyalgia; HF HRV = high frequency heart rate variability; PEP = pre-ejection period; SCL = skin conductance level. * = significant difference from baseline.

Group results of the response to the nociceptive stressor are shown in Fig. 3. The average scores (% correct) on the PVSAT test are shown in Table 1. During the ANS session, the scores were significantly different among groups (*p* = 0.04), with both the knee OA (*p* = 0.02) and FM (*p* = 0.046) groups having a significantly lower score than the control group. In the nociceptive session, the scores were not significantly different among the three groups (*p* = 0.1).

There was a significant reduction in HF HRV in the control group during the mental stressor compared to baseline (*p* = 0.006). In contrast, HF HRV was not significantly different during the mental stressor compared to baseline in the knee OA (*p* = 0.78) or FM (*p* = 0.13) groups.

PEP was significantly shorter during the mental stressor compared to baseline in the control (*p* = 0.008), knee OA (*p* = 0.01), and FM (*p* = 0.01) groups.

There was a significant increase in SCL in all three groups during the mental stressor in comparison to baseline (all *p* < 0.001).

There was a significant reduction in tonic heat pain rating in the control group after the mental stressor compared to baseline (*p* = 0.009). In contrast, tonic heat pain ratings were not significantly different following the mental stressor compared to baseline in the knee OA (*p* = 0.08) or FM (*p* = 0.38) groups.

## 4 Discussion

This is one of the first studies to show impaired ANS function in people with knee OA, both at rest and in response to acute nociceptive and mental stress. People with knee OA consistently showed reduced parasympathetic activity at rest and impaired withdrawal during acute stress. Less consistent findings were evident for measures of sympathetic function, with some evidence of an impaired central sympathetic response to acute nociceptive stress. The hypoalgesic effect of the two stressors was also diminished in both the knee OA and FM groups.

### 4.1 ANS function at rest

Reduced parasympathetic activity during resting conditions, reflecting reduced vagal tone, is a common finding in chronic pain conditions (Tracy et al., 2016). This is one of the first times impaired vagal function has been shown in an OA population and, while the findings need to be considered preliminary due to the small sample size, they support our previous work in the same population (Bossenger et al., 2023). Low resting vagal tone is associated with a number of other chronic conditions, including cardiovascular disease (Thayer and Lane, 2007), diabetes (Jaiswal et al., 2013), mood disorders (Agelink et al., 2002), and sleep dysfunction (Spiegelhalder et al., 2011). While causality cannot be determined from these findings, reduced vagal tone may put people at increased risk of chronic pain and other long-term conditions, including knee OA. For example, Courties et al. (Courties et al., 2017) discuss evidence of anti-inflammatory effects of parasympathetic fibres within joint tissue and the potential of vagus nerve stimulation to reduce systemic inflammation in inflammatory chronic pain conditions. There is also strong interaction and overlap of autonomic and nociceptive centres within the brainstem, including indirect vagal input into the nucleus tractus solitaris. The nucleus tractus solitaris projects to multiple other brainstem regions involved in nociceptive modulation (Krahl and Clark, 2012, Millan, 2002). Thus, impairment of these anti-inflammatory and tonic nociceptive inhibitory mechanisms through reduced resting vagal tone may be contributing to pain in people with OA.

The FM group showed higher baseline sympathetic drive through increased SCL but this was not significant in the knee OA group. Electrodermal activity reflects sympathetic cholinergic neurotransmission (Illigens and Gibbons, 2009). Increased sympathetic activity causes the release of the endogenous catecholamines adrenaline and noradrenaline, which are known to influence OA-related pain (Sohn et al., 2021). The influence of noradrenaline on joint tissue specifically is catabolic and pro-inflammatory, and therefore potentially contributes to increased pain in FM (Courties et al., 2017, Sohn et al., 2021). In contrast to the SCL findings, PEP values of both chronic pain groups were equivalent to controls at rest. This is consistent with some previous studies in FM (Barakat et al., 2012, Contreras-Merino et al., 2022), although others (Reyes del Paso et al., 2011, Reyes del Paso et al., 2010, Reyes del Paso et al., 2021) have found myocardial contractility to be lower in people with FM at rest compared to pain-free controls. While both SCL and PEP are under sympathetic control, electrodermal activity and cardiac contractility are mediated by different neurotransmitters and branches of the SNS, and therefore may function differently. We found no evidence of altered resting function in the central SNS in either group.

### 4.2 Response to stress

The control participants showed a substantial reduction in HF HRV during exposure to both of the acute stressors. This vagal withdrawal follows previous studies that have shown similar responses using both acute mental (Reyes del Paso et al., 2010, Xie et al., 2017, Tanosoto et al., 2012) and nociceptive (Wirch et al., 2006, Mourot et al., 2009) stimuli in healthy populations. However, significant vagal withdrawal did not occur during either of the stressors in the knee OA or FM groups. This supports one previous study that showed impaired HF HRV modulation in response to mental stress in people with FM (Reyes del Paso et al., 2010), but is the first time it has been shown in an OA population. The lower resting vagal tone in the chronic pain groups likely limits the ability of the parasympathetic system to respond appropriately to stressors, creating a floor effect that impedes the modulatory capacity of the system (Laborde et al., 2018).

It is well established that acute psychological stress, including mental arithmetic (Mezzacappa et al., 2001, Ring et al., 2000), raises beta adrenergic activity, resulting in shortened PEP and increased cardiac contractility (Brindle et al., 2014). A shortened PEP was evident in the control group during both stressors. However, a significant shortening in PEP was only evident in response to the mental stressor in the knee OA and FM groups, although the lack of significant findings during the nociceptive stressor session may have been related to sample size and increased variability in the chronic pain groups. Previous research has shown that modulation of PEP in response to different stressors is intact in people with FM (Reyes del Paso et al., 2021) and knee OA (Bossenger et al., 2023), and we therefore recommend further investigation to clarify cardiac sympathetic reactivity to different stressors in people with knee OA. SCL increased significantly in all three groups during both acute mental and nociceptive stress. This was expected in the control group (Fechir et al., 2008) but went against our hypothesis of a blunted response in the two chronic pain groups. Our findings therefore provide evidence that the cutaneous sympathetic response to stress is intact in people with knee OA and FM, even though resting SCL may be elevated.

In accordance with our hypothesis, the control group showed significantly reduced tonic heat pain ratings immediately following the acute mental and nociceptive stressors. Previous studies in pain free people support these findings of mental stress-induced hypoalgesia (Drummond et al., 2001, Terkelsen et al., 2004, Yilmaz et al., 2010). While this is the first time the nociceptive effects of acute mental stress have been examined in an OA population, an impaired hypoalgesic effect of mental arithmetic has been shown previously in people with FM (Drummond and Willox, 2013, Thieme et al., 2006) and other chronic pain conditions (Drummond and Willox, 2013). The hypoalgesic effect of nociceptive input is also well known and has previously been shown to be altered or more variable in people with chronic pain (Lewis et al., 2012), including FM (Julien et al., 2005, Potvin and Marchand, 2016, Lautenbacher and Rollman, 1997) and OA (Arendt-Nielsen et al., 2010, Kosek and Ordeberg, 2000).

Mental and nociceptive stress-induced hypoalgesia in healthy people can be activated by several means, including opioid and non-opioid pathways (Yilmaz et al., 2010, Flor et al., 2002, Bandura et al., 1987) and the cardiovascular system in the form of hypertension-induced hypoalgesia (Bruehl and Chung, 2004, Ghione, 1996). Altered ANS function in response to stress is therefore likely to not only impact the cardiovascular response to stress but also other systems that the ANS and cardiovascular systems interact with, including the nociceptive system. As some evidence for this, a reduced cardiovascular response to a nociceptive stressor has been linked with impaired inhibition of the nociceptive system in people with FM (Chalaye et al., 2014). Therefore, the impaired ANS response to stress may directly contribute to reduced stress-induced hypoalgesia through a diminished ability to activate nociceptive inhibitory mechanisms.

### 4.3 Limitations

This study is not without limitations. Participant recruitment was impacted by COVID-19 restrictions and therefore the analyses may be under-powered for some variables. Additionally, previously undiagnosed cardiac issues in two participants resulted in the loss of further ECG data. Therefore, the findings must be considered preliminary until confirmed in a larger study. The PVSAT is a computerised version of the PASAT, requiring the use of a mouse and laptop. A degree of dexterity to coordinate the mouse cursor onscreen is required, and age and hand-eye coordination may have made the task more difficult for the knee OA group, resulting in their lower scores. PVSAT performance results were lower during the ANS session in all three groups, possibly due to the need to remain still in a recumbent position while completing the task. There is some evidence that both older age (e.g., Jandackova et al., 2016) and higher levels of BMI (e.g., Karason et al., 1999) are associated with lower HF HRV, which may have impacted our findings in the knee OA group. Future, fully powered studies, should control for these variables and the multiple comparisons undertaken in the statistical analyses.

### 4.4 Conclusion

The findings provide some initial evidence of impaired autonomic function in people with knee OA, including reduced vagal tone at rest and impaired vagal withdrawal in response to acute stress. There was also some, albeit inconsistent, evidence of increased sympathetic function at rest and a blunted sympathetic response to stress. The coincident impairment in the normal hypoalgesic effect of acute stress suggests that further research is undertaken to determine if interventions that improve ANS function can alter nociceptive system function and the experience of pain in people with knee OA.

### CRediT authorship contribution statement

**Neil R. Bossenger:** Conceptualization, Methodology, Investigation. **Gwyn N. Lewis:** Conceptualization, Methodology, Supervision. **David A. Rice:** Conceptualization, Methodology, Resources, Supervision. **Daniel Shepherd:** Conceptualization, Methodology, Resources, Supervision.

## Declaration of Competing Interest

The authors declare that they have no known competing financial interests or personal relationships that could have appeared to influence the work reported in this paper.
